# A Suggestion—Journalistic

**Published:** 1883-06

**Authors:** 


### A Suggestion—Journalistic.

The journalistic interests of the dental profession of the country have assumed such an importance and magnitude, as to warrant associate and conjoined consideration on the part of those now immediately connected with and conducting its affairs.

In all great interests, it is generally conceded, that in union and co-operation there is strength. With this fact in mind, it is here suggested that there be held at Niagara Falls, at the time of the meeting of the American Dental Association, a meeting of all the editors of dental pereodicals and papers in the country, with the view at least of social converse, and considering so far as may be practicable matters of general journalistic interest; and out of this perhaps might arise a permanent organization. Such a meeting would be pleasant and doubtless profitable. Brethren of the tripod, do you homologate? If so will somebody name the day, place and hour.

EDITOR DENTAL REGISTER:—Will you, please, tell me the meaning of D.M.D., the degree granted by the Harvard School? I have (the Lord knows) enough D's to my name, viz: M.D., D.D.S. Some say it means, Doctor of Mechanical Dentistry. Is that so? *

THE stiffest-necked boy in Schuylkill County lives in Pottsville. He fell off a farm sleigh and the runner passed over his neck. When a crowd from the corner store ran to pick up the supposed corpse, the boy scrambled to his feet and ran after the sleigh.

